# Bioprotective Respirator Assembled by Defective Carbon Nitride for Long‐Term Light Triggered Health Protection

**DOI:** 10.1002/advs.202403098

**Published:** 2024-06-19

**Authors:** Zhenxing Zeng, Qi Zhang, Fei Ye, Xueming Dang, Xin Jiang, Guochun Lv, Xiaojing Wang, Hong Peng, Dexin Fang, Hong Xiao, Yanzong Zhang, Ganxue Wu, Jie Mao, Munir Ahmad, Shihuai Deng

**Affiliations:** ^1^ College of Environmental Sciences Sichuan Agricultural University Chengdu 611130 P. R. China; ^2^ Sichuan Provincial Engineering Center of Agricultural Environmental Pollution Control Chengdu 611130 P. R. China; ^3^ Hebei Key Laboratory of Applied Chemistry School of Environmental and Chemical Engineering Yanshan University Qinhuangdao 066004 P. R. China; ^4^ Key Laboratory of Industrial Ecology and Environmental Engineering (Ministry of Education) School of Environmental Science and Technology Dalian University of Technology Dalian 116024 P. R. China; ^5^ School of Environment and Energy Jiangxi Modern Polytechnic College Nanchang 330095 P. R. China; ^6^ Research Center for Eco‐Environmental Sciences Chinese Academy of Sciences Beijing 100085 P. R. China; ^7^ Institute of Carbon Neutrality Zhejiang Wanli University Ningbo 315100 P. R. China

**Keywords:** bio‐protective respirator, defective g‐C_3_N_4_ nanosheets, long term protection, pathogen inactivation, photocatalysis

## Abstract

Wearing face masks is the best way to stop the spread of respiratory infections. However, if masks are not sterilized, changing them too frequently can actually increase the risk of cross‐contamination. Herein, the construction of an antipathogen photocatalytic mask with carbon vacancy‐modified carbon nitride nanosheets (g‐C_3_N_4_‐V_C_ Ns) coated on the non‐woven fabrics of the out layer of the mask, offering effective and long‐term protection against damaging pathogens when exposed to light is reported. The introduced carbon vacancies are found capable of creating energy‐disordered sites and inducing energetic electric force to overcome the Coulomb interactions between electron‐hole pairs, thus promoting the electron‐hole separation to achieve a high generation of reactive oxygen species (ROS). Thanks to its high activity in generating ROS upon exposure to light, the as‐prepared photocatalytic mask shows high pathogen sterilization performance. This, in turn, prolongs the mask's protective lifetime, decreases the need for regular replacement, and decreases medical waste production. The work demonstrated here opens new viewpoints in designing pathogens biocidal protective devices for health protection, offering significant promise in specific environment self‐protection.

## Graphical abstract

Carbon vacancy installation is found capable of creating energy disordered landscape to overcome the Coulomb interactions between electron‐hole pairs, thus promoting the electron‐hole separation to achieve a high generation of reactive oxygen species (ROS). Thanks to its high activity in generating ROS upon exposure to light, the as‐prepared photocatalytic mask shows high pathogen sterilization performance.

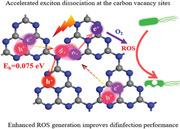

## 1 Introduction

In recent years, personal health protection has gained substantial attention, due to global virus outbreaks caused by extremely infectious respiratory pathgens like COVID‐19, resultanting in innumerable death, and suffering.^[^ ^1^, ^2^, ^3^, ^4^ ^]^ Although some of the diseases caused by pathogen aerosol infection can be cured, the irreversible damage to our human body may cause a series of sequelae that heavily affect our daily life.^[^ ^5^, ^6^, ^7^ ^]^ To date, wearing a face mask is the most effective and direct way to capture and interrupt pathogens transmission, thus protecting ourselves from airborne microorganism infection.^[^ ^8^, ^9^ ^]^ Nevertheless, pathogens are merely physically captured and intercepted by the mask rather than being killed. However, these pathogens, with sustained infective activity, still pose a high risk of causing infection. Shortening the wear time or increasing the frequency of replacing face masks, for example, every 4 h for commercial medical face masks, has been considered as a potential way to decrease the risk of infection.^[^ ^10^, ^11^ ^]^ However, such a high replacement frequency not only increases the risk of cross‐contamination and pathogen spreading but, even worse, can lead to the generation of large amounts of infectious medical waste that poses severe threats to the ecological environment.^[^ ^12^ ^]^ Thus, the exploration of bio‐protective masks with pathogen inactivation ability is highly desired to achieve long‐term antimicrobial protection from infective pathogen aerosol.

**Scheme 1:**
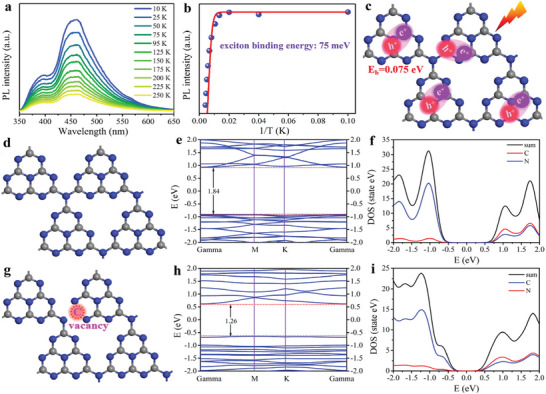
a) Temperature‐dependent PL spectra and b) the correspondent integrated PL emission intensity of carbon nitride. c) Illustration of photo‐induced excitons in carbon nitride. d) Structure models of (d) g‐C_3_N_4_ Ns and (g) g‐C_3_N_4_‐V_C_ Ns. e,f) Calculated band structures and h,i) corresponding DOS for g‐C_3_N_4_ Ns and g‐C_3_N_4_‐V_C_ Ns, respectively.

Photocatalysis in recent years has been shown as a promising way for pathogen sterilization.^[^ ^13^, ^14^ ^]^ The introduction of photocatalysts onto the surface of the face masks to construct a pathogen sterilization mask may serve as an alternative to achieve the aforementioned hypothesis. Since the photocatalytic sterilization is reported to follow the reactive oxygen species (ROS) oxidation process,^[^ ^15^, ^16^ ^]^ the ROS generation activity of the photocatalyst serves as the key to determining the bio‐protective performance of the photocatalytic mask. Various photocatalysts with efficient ROS production have been explored, while most of them are metal‐based having poor binding interactions with the non‐woven fabrics of the mask, making them prone to dropping off. This leads to a loss of pathogen inactivation efficacy in the mask.^[^ ^17^, ^18^, ^19^ ^]^ Metal‐free carbon nitride (g‐C_3_N_4_) has been shown as a promising photocatalyst for microorganism sterilization because of its several merits such as visible light response, easy structure manipulations, and easy availability.^[^ ^20^, ^21^, ^22^ ^]^ However, unlike the transition metal‐based semiconductors with a strong screening effect, the photo‐generated electrons and holes in polymeric material exit in the form of bound electron‐hole couples (known as Frenkel exciton or singlet exciton) with strong Coulomb interactions due to the low dielectric constant and large wave functions overlap between electron and hole.^[^ ^23^, ^24^, ^25^ ^]^ Such a strong Coulomb interaction with binding energy tested to be as high as 75 mV shown in **Scheme** **1**, leading to poor ROS generation performance as a result of fast electron‐hole recombination. Various strategies aiming at boosting the electron‐hole separation have been reported, while limited ROS generation performance enhancement is achieved as most of them ignored the Coulomb interactions that should actually be taken into consideration.^[^ ^26^, ^27^, ^28^, ^29^ ^]^

Given that the energy‐disordered landscape can offer a high build‐in electric force to facilitate the electron‐hole couple separation,^[^ ^30^, ^31^, ^32^ ^]^ we deduce that the creation of defects sites, such as vacancy, may also induce local electronic state redistributions to facilitate electron‐hole separation. Specifically, the build‐in electric force provided by the defect site should be at least larger than the Coulomb force of the electron‐hole, otherwise, electron‐hole separation will not happen. DFT calculations (Scheme 1) show that the lowest unoccupied molecular orbital (LUMO) and highest occupied molecular orbital (HOMO) levels of heptazine‐based melon chains are calculated to be ≈0.93 and −0.91 eV, while the LUMO and HOMO levels are ≈0.63 and −0.63 eV after the modification with carbon vacancy. These results demonstrate that carbon vacancy modification can induce local energy structure disorder and, more importantly, provide energetic electric force for exciton dissociation. Density of state (DOS) calculations indicate that both materials possess typical semiconductor characteristics, as the Femi level is found located between the HOMO and LUMO. In addition, adjusting the amount of introduced vacancy is possible to control the vacancy‐induced energy‐disordered interfaces, which is beneficial for promoting electron‐hole separation.

In this study, we propose carbon vacancy modification of carbon nitride nanosheets (g‐C_3_N_4_ Ns) to create locally distorted energy sites, thus increasing electron‐hole separation and attaining high photocatalytic ROS creation activity. Based on the carbon vacancy‐modified carbon nitride nanosheets (g‐C_3_N_4_‐V_C_ Ns), we have developed a bio‐protective face mask with pathogen sterilization ability to improve the protection performance and prolong the protective time. The constructed face mask demonstrates excellent long‐term health protection under light‐triggered conditions in pathogen environments.

## 2 Results and Discussion

**Figure 1:**
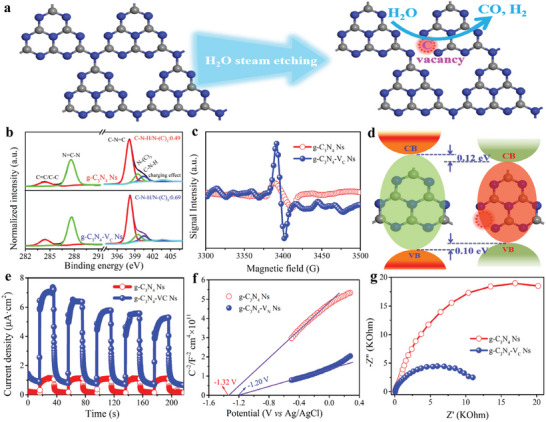
a) Schematic illustration of steam‐mediated carbon vacancy installation in carbon nitride. b) XPS C1s, N1s, and c) EPR spectra of g‐C_3_N_4_ Ns and g‐C_3_N_4_‐V_C_ Ns. d) Illustration of energy band structures of g‐C_3_N_4_ Ns and g‐C_3_N_4_‐V_C_ Ns. e) Transient photocurrent, f) Motto–Schottky plots, and g) EIS spectra of g‐C_3_N_4_ Ns and (b) g‐C_3_N_4_‐V_C_ Ns.

The carbon vacancy‐modified carbon nitride nanosheets (g‐C_3_N_4_‐V_C_ Ns) are fabricated via a classical steam reforming strategy according to the previously reported method with some modifications.^[^ ^33^, ^34^ ^]^ During the high‐temperature steam treatment, the water molecule can etch the carbon atoms of carbon nitride, accompanied by the release of water gas, as illustrated in **Figure** **1**a. Element analysis from the XPS shows that the surface C/N ratio decreased from initial 0.87 to 0.84 (Table S1, Supporting Information), confirming the selective removal of carbon atoms by steam. It is still necessary to uncover which site of carbon atom in the carbon nitride skeleton has been etched, thus XPS tests are performed and the C 1s and N 1s of g‐C_3_N_4_ Ns and g‐C_3_N_4_‐V_C_ Ns are carefully analyzed. As shown in Figure 1b, the N‐(C)_3_ assigned peak area is obviously decreased after the steam etching treatment, while accompanied by the significantly increased C‐N‐H/N‐(C)_3_ ratio from 0.49 to 0.69 from the N 1s spectra, suggesting the carbon lost at the N‐(C)_3_ site. The successful introduction of carbon vacancies is further confirmed by the solid state EPR, where a prominent EPR peak with a g value ≈2.0 is found (Figure 1c).^[^ ^35^ ^]^ It should be noted here that the water steam and released water gas during the high‐temperature treatment may serve as pore‐creation reagents to create pores on the nanosheets, as supported by the observations of abundant pores on the nanosheets of g‐C_3_N_4_‐V_C_ Ns from the SEM and TEM images shown in Figure S1 and S2 (Supporting Information). The creation of abundant pores on the nanosheets doesn't alert the typical carbon nitride skeleton a lot, since both materials give comparable XRD patterns, FT‐IR, and XPS spectra (Figure S3, Supporting Information; Figure 1b).

It is still necessary to check if the disordered energy landscape caused by the carbon vacancies installation is powerful enough to overcome the exciton binding energy for exciton dissociation. As shown in Figure S4 (Supporting Information), the introduction of carbon vacancies leads to the optical property change, where the energy band gap decreased from 2.71 eV of g‐C_3_N_4_ Ns to 2.49 eV of g‐C_3_N_4_‐V_C_ Ns. Besides the energy band gap decrement, a secondary light adsorption edge in the visible region can be found, attributing to the modification of carbon vacancies that alert the local electronic state distributions. Motto–schottly plots show that the flat band positions of g‐C_3_N_4_ Ns and g‐C_3_N_4_‐V_C_ Ns are respectively tested to be 1.32 and −1.20 eV, implying that both materials are energetically capable of driving the oxygen reduction reactions for reactive oxygen species (ROS) production.^[^ ^36^ ^]^ Based on the above analysis, the proposed energy band structures of these two materials are illustrated in Figure 1d, in which one can easily find that the energy band distortion caused by the vacancy installation is more than 0.10 eV. Such a high energy band distortion may lead to the formation of a build‐in electric field with a powerful driving force (0.10 eV) to overcome the binding energy of photo‐induced exciton in carbon nitride (0.075 eV), in line with the DFT calculations demonstrated in Scheme 1. As the electric force of the build‐in field is much higher than the exciton Coulomb interaction, exciton dissociation at the vacancy site is therefore expected, and the proposed exciton dissociation at the carbon vacancy site is illustrated in Figure S5 (Supporting Information).

Since the exciton tends to dissociate around the vacancy site, the charge carriers in g‐C_3_N_4_ Ns and g‐C_3_N_4_‐V_C_ Ns may therefore show different behavior.^[^ ^24^ ^]^ As depicted in Figure S6 (Supporting Information), g‐C_3_N_4_‐V_C_ Ns gives a much lower PL intensity than the counterpart material, revealing the decreased singlet exciton population.^[^ ^24^, ^37^ ^]^ Time‐resolved PL spectra (tested at the corresponding emission wavelength) show that g‐C_3_N_4_‐V_C_ Ns exhibits an shortened lifetime with respect to that of g‐C_3_N_4_ Ns (Figure S7 and Table S2, Supporting Information). Since the PL originates from the singlet exciton recombination, the reduced singlet exciton lifetime suggests the promoted singlet exciton dissociation in the carbon vacancy case. The dissociation of exciton around the vacancy site will lead to the generation of free charge carriers, while spatial separation of these charge carriers will subsequently proceed driving by the buil‐in electric field. This can be further validated by the transient photocurrent shown in Figure 1e, in which the current density of g‐C_3_N_4_‐V_C_ Ns is more than four times higher than the reference material of g‐C_3_N_4_ Ns. As a result of the accelerated charge separation, a much higher free electron concentration is observed from the Motto‐Schottky plots, where the curve slope of g‐C_3_N_4_‐V_C_ Ns is several times lower than that of g‐C_3_N_4_ Ns. Electrochemical impendence spectra (EIS) show that g‐C_3_N_4_‐V_C_ Ns displays a much smaller semi‐cycle radius than the counterpart material of g‐C_3_N_4_ Ns, suggesting the reduced charge transportation resistance due to the exciton dissociation that allows the fast charge transfer. This can be further confirmed by the increased electron lifetime shown in the Bode phase plots (Figure S8, Supporting Information). Based on the above analysis, one can conclude that carbon vacancy modification can create energy‐disordered sites in carbon nitride and provide powerful built‐in electric force to facilitate the exciton split, leading to enhanced charge separation.

**Figure 2:**
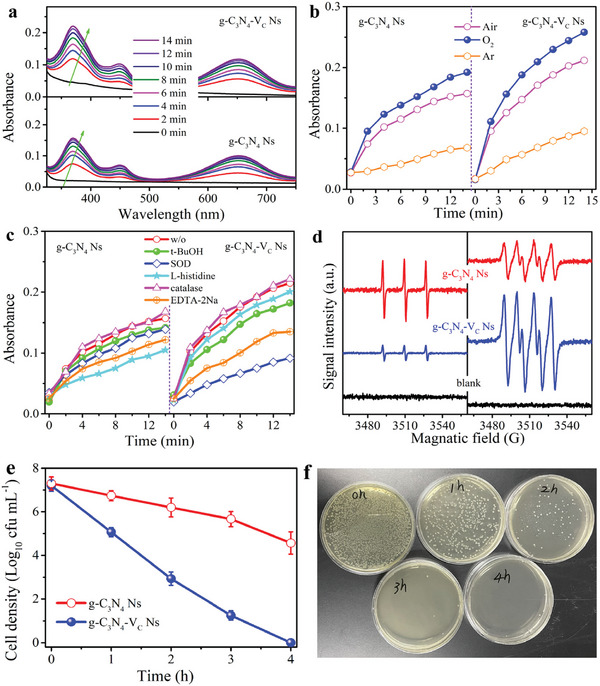
a) Time‐dependent absorption spectra of TMB oxidation over g‐C_3_N_4_ Ns and g‐C_3_N_4_‐V_C_ Ns in air condition. b) The absorbance of TMB oxidation monitoring at 651 nm at different atmospheric conditions (air, Ar, and O_2_). c) The absorbance changes of TMB oxidation at 651 nm in the presence of different radical scavengers. d) EPR spectra of g‐C_3_N_4_ Ns and g‐C_3_N_4_‐V_C_ Ns in the presence of TEMP (left) and DMPO (right). e) Photocatalytic E‐coli inactivation performance over g‐C_3_N_4_ Ns and g‐C_3_N_4_‐V_C_ Ns under visible light (λ > 420 nm) irradiation. f) Digital photos of time profile photocatalytic E‐coli inactivation on g‐C_3_N_4_‐V_C_ Ns with different dilution ratios.

With improved charge separation and prolonged free electron lifetime, a higher photocatalytic oxygen reduction reaction performance for ROS generation is expected. Here, the typical TMB oxidation reaction is employed as a probe reaction to identify and quantify the generated ROS, since almost all oxidative species including photo‐generated holes are capable of oxidizing TMB to give a typical blue color.^[^ ^38^, ^39^ ^]^ As displayed in **Figure** **2**a, both materials show two distinguish light absorbance peaks ≈380 and 650 nm, attributed to the oxidized intermediates derived from TMB oxidation. In addition, the light absorbance of g‐C_3_N_4_‐V_C_ Ns at both 380 and 651 nm is much higher than the counterpart material of g‐C_3_N_4_ Ns, indicating the enhanced ROS production rate after carbon vacancy modification. As illustrated in Figure 2b, both materials show prominent TMB oxidation characteristics in different atmospheres of Ar, Air, and O_2_ (the TMB oxidation performance follows the trend of O_2_ > Air > Ar), demonstrating that the generated ROS are evolved from the O_2_ activation process.

To achieve this, it is essential to recognize the type of generated ROS, thus radical capture experiments are performed. Here, SOD, L‐histidine, t‐BuOH, catalase, and EDTA‐2Na are adopted as probes to identify the reactive species of O_2_^·−^, ^1^O_2_, ·OH, H_2_O_2_, and photo‐induced holes. As depicted in Figure 2c, the addition of L‐histidine into reaction system largely quenched the TMB oxidation performance of g‐C_3_N_4_ Ns, while TMB oxidation activity decrement with the addition of other three additives (SOD, t‐BuOH and catalase) is not that far than that of L‐histidine, indicating that ^1^O_2_ is the main reactive species contributing to the TMB oxidation in the g‐C_3_N_4_ Ns system. For g‐C_3_N_4_‐V_C_ Ns, the introduction of SOD into the reaction system significantly screens the TMB oxidation activity, while no obvious TMB oxidation decrement can be found with the addition of other three radical captures, demonstrating that O_2_^·−^ is the main radical generated in the system. It is worth noticing here that the addition of an excess amount of SOD into the system cannot fully screen the TMB oxidation of g‐C_3_N_4_‐V_C_ Ns, which should attribute to the fact that photo‐induced hole is also capable of oxidizing TMB, as supported by the obvious TMB oxidation activity loss with the addition of EDTA‐2Na as hole capture into the reaction system. The type of generated radicals for both materials are further confirmed by using EPR tests, where 5, 5‐dimethyl‐1‐pyrroline‐N‐oxide (DMPO) and 2, 2, 6, 6‐tetramethyl‐4‐piperidone (TEMP) are adopted as testing probes to detect the generated O_2_^·−^ and ^1^O_2_. As illustrated in Figure 2c, a typical triplet signal with the intensity of 1:1:1 can be observed for the g‐C_3_N_4_ Ns system when using TEMP as the testing probe, confirming that ^1^O_2_ is the main reactive species of g‐C_3_N_4_ Ns. As a reference, a rather weak peak intensity is found for g‐C_3_N_4_‐V_C_ Ns, confirming the negligible ^1^O_2_ generation for g‐C_3_N_4_‐V_C_ Ns. When using DMPO as the testing probe, a classic quaternate‐shaped signal with signal intensity of 1:1:1:1 can be found for g‐C_3_N_4_‐V_C_ Ns, confirming the generation of O_2_^·−^. While the quaternate signal referring to O_2_^·−^ is also observed for g‐C_3_N_4_ Ns, the peak intensity is much lower than that of g‐C_3_N_4_‐V_C_ Ns, indicating the rather low amount of generated O_2_^−^.

The photocatalytic activity of as‐prepared powder form materials is firstly evaluated using the bacteria inactivation reactions, where E‐coli with a concentration ≈10^7^ cfu mL^−1^ is used as the simulated pathogen. As shown in Figure 2e, the bacteria concentration decreased from its initial 10^7^ cfu mL^−1^ to ≈10^5^ cfu mL^−1^ for the g‐C_3_N_4_ Ns‐based catalytic system after 4 h visible light irradiation, while complete bacteria inactivation is achieved when using g‐C_3_N_4_‐V_C_ Ns as photocatalyst. The time‐dependent bacteria inactivation in Figure 2f shows the gradual decrement of E‐coli colony, reflecting the photocatalytic bacteria killing procedure. The much faster water disinfection of g‐C_3_N_4_‐V_C_ Ns originated from the improved ROS generation amount as the result of vacancy‐triggered exciton dissociation, in agreement with the result shown in Figure 1.

**Figure 3:**
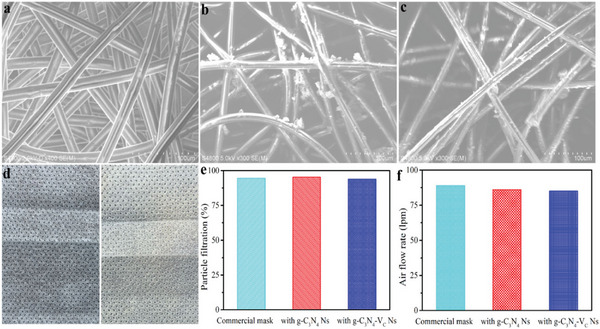
SEM images of the out‐layer non‐woven fabrics a) without any modification, and functionalized with g‐C_3_N_4_ Ns and c) g‐C_3_N_4_‐V_C_ Ns. d) Digital photos of the non‐woven fabrics modified with g‐C_3_N_4_ Ns (left) and g‐C_3_N_4_‐V_C_ Ns (right). e) Particle filtration and f) air flow rate of the commercial mask, and g‐C_3_N_4_ Ns and c) g‐C_3_N_4_‐V_C_ Ns functionalized respirators.

In this work, the carbon‐vacancy modified carbon nitride is designed as a high ROS production functional material for the construction of pathogen inactivation respirator. To this end, a simple modified spraying method uses a homemade sprayer to spray the catalyst dispersion into the non‐woven fabrics. As shown in **Figure** **3**, the non‐woven fabrics of the commercial mask show a clean and smooth surface, while after spraying the materials of g‐C_3_N_4_ Ns and g‐C_3_N_4_‐V_C_ Ns, the fabrics become rough, and particles can be found distributed on the surface of the fabrics. The observations of catalyst particles on the surface of fabrics demonstrate the successful construction of catalyst‐modified fabrics, as supported by the obvious color changes of the non‐woven fabrics when compared to the unmodified ones (Figure 3d). The modified non‐woven fabrics are then sewed up to the mask and used as the outer layer of the mask, obtaining the pathogenic inactivation functional mask (Figure S10, Supporting Information). The modification of the outer layer of non‐woven fabrics may affect the protective performance of the face mask, thus the breathability and particle filtration properties of the as‐constructed masks are further investigated. As shown in Figure 3e, no obvious particle rejection performance change can be found, suggesting the negligible filtration ability alert after the modification of the fabric with carbon nitride. In addition, a negligible air flow rate decrement under the same testing pressure is tested for the functionalized mask compared to the commercial one, ruling out the breathability effect via carbon nitride modification.

**Figure 4:**
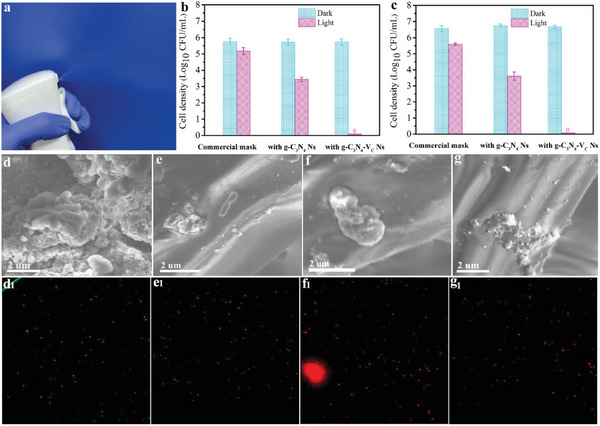
a) Simulated bacterial aerosols over a homemade device. Survival b) E‐coli and c) S‐aureus cells on the outer layer of functionalized respirators after 45 min visible light irradiation. SEM and corresponding fluorescence microscopic images of E‐coli cells on the out layer of the g‐C_3_N_4_‐V_C_ Ns functionalized respirator after being illuminated by the bacteria‐loaded respirator at different times of d) 0 min, e) 15 min, f) 30 min, and g) 45 min.

Since the vacancy‐installed carbon nitride nanosheets can drive the oxygen activation reactions for ROS generation under light irradiation, the loading of these materials onto the non‐woven fabrics may endow the mask with light‐triggered pathogens inactivation ability. During the mask‐wearing period, the photocatalyst on the layer of non‐woven fabrics of the mask can be excited to generate charge carriers, after undergoing the vacancy‐triggered exciton split and electron‐hole spatial separation, the free electrons are capable of driving the oxygen reduction reaction to generate ROS. The biocidal process is supposed to happen as the produced ROS is highly biological toxicity to pathogens, which surely could reduce the possibility of microorganisms passing through the mask to cause infection. With the biocidal ability, the functionalized mask use time may therefore prolonged, which reduces the mask replacement frequency and thus decreases the medical waste. Here, a homemade atomizer is used to generate pathogens aerosol, and a fixed amount of E‐coli or S‐aureus with an initial concentration of ≈10^6^ cfu mL^−1^ (**Figure** **4**a) is used as the target pathogen, to evaluate the protection performance of the as‐prepared photocatalytic mask. As shown in Figures 4b,c, after subjecting the mask to visible light irradiation for 45 min, negligible bacteria concentration decrement is found for the commercial mask, confirming the commercial mask's non‐bacterial killing ability. While notable bacteria concentration reduction is achieved for the g‐C_3_N_4_ Ns loaded mask, attributing to the possible generation of ^1^O_2_ that can inactivate bacteria cells. As a result of the high O_2_^·−^ generation amount and higher oxidative ability than that of ^1^O_2_, the g‐C_3_N_4_‐V_C_ Ns functionalized mask shows complete bacteria inactivation within 45 min, demonstrating the excellent bacteria‐killing performance of the as‐prepared photocatalyst mask. Other materials that are capable of driving the oxygen activation reaction for ROS generation under light irradiation, can be used for the construction of a photocatalytic mask (Figure S9, Supporting Information).

It is necessary to investigate the pathogens's biocidal process on the catalyst‐modified non‐woven fabrics of the outer layer of the mask, the morphology of bacteria on the fabrics after being illuminated for different times is visualized by SEM. As shown in Figure 4d, without light irradiation, bacteria on the modified fabrics give a plump bacillar morphology with a smooth but intact membrane surface. Pores can be observed distributed on the bacteria membrane after 15 min photocatalytic reactions (Figure 4e), attributing to the generated ROS that can destroy the proteins and phospholipids of the membrane. Prolonged irradiation leads to the generation of more ROS that can further create pores and enlarge the pore size of the membrane that cannot be repaired by the bacteria itself, leading to the leakage of cytoplasm out of the cell through the pores on the cell membrane. As a result, the bacteria on the fabrics show a wizened shape (Figure 4f). Complete bacteria destruction is achieved after 45 min, as only small parts of the bacteria remnants can be found in the SEM image in Figure 4g. Complete E‐coli bacteria inactivation can be further supported by the fluorescence microscopic images shown in Figure 4d_1_–g_1_. Moreover, the typical carbon nitride skeleton remains unchanged (Figure S11, Supporting Information), revealing the high stability of the material.

**Figure 5:**
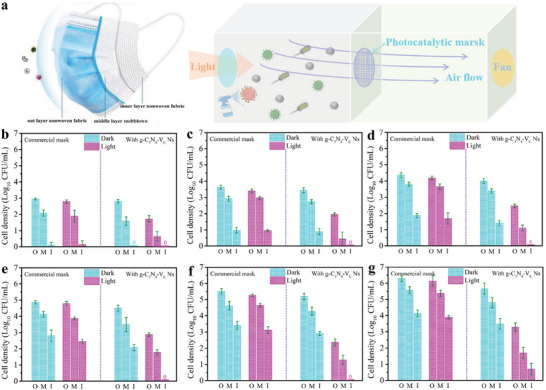
a) Illustration of the simulated pathogenic microorganism protection of carbon nitride functionalized respirator in a homemade reactor simulating the breathing process (note: the aerosols are continuing to be produced during the tests). Survival bacteria cells on out layer (abbreviated as O), middle layer (abbreviated as M), and inner layer (abbreviated as I) after being illuminated b) 1 h, c) 2.0 h, d) 3.0 h, e) 4.0 h, f) 5.0 h, and g) 6.0 h.

It should be noted here that the pathogens aerosol transmission is quite sensitive to the airflow,^[^ ^9^ ^]^ thus it is necessary to build a reactor simulating the human breathing process to make the testing results more convincible. As shown in **Figure** **5**a, a two‐chamber reactor separated by the photocatalytic respirator is proposed, where an electric fan is installed and used to control the airflow directions simulating the breathing process. Furthermore, a homemade sprayer is employed to generate pathogens containing aerosol simulating the pathogens transmitting environment. For safety considerations, the mask protection experiments are conducted on a Clean Bench and aerosol‐transmitting S‐aureus is employed as an infective pathogen. A xenon lamp is used as a light source and filters are used to the control the output light wavelength. As illustrated in Figure 5, without light irradiation the surviving bacteria concentration on the g‐C_3_N_4_‐V_C_ Ns functionalized respirator is almost comparable to the commercial one, ruling out the effect of carbon nitride material on the bacteria living. While the bacteria concentration on either the outer layer, middle layer, or inner layer of the mask increases over time, ascribed to the continuous generation of bacteria aerosol on the left side of the reactor, after switching on the light, the bacteria concentration on the commercial mask is slightly lower than in dark conditions. This could be due to the photothermal effect contributing to bacteria inactivation.^[^ ^40^, ^41^, ^42^ ^]^

It is worth noticing here that it is the middle layer of melt‐blown fabrics that contributes to the mask filtration, this is why the bacterial concentration on the inner layer is far less than the outer and middle layers. However, the commercial mask can only last for one hour in such an aerosol environment, beyond which pathogen with a higher concentration on the mask may pass through the three layers, leading to the infection. In other words, mask replacement is necessary when the commercial mask is worn for one hour in such a high bacteria aerosol environment. Otherwise, infection is going to happen. As for the g‐C_3_N_4_‐V_C_ Ns functionalized respirator, even if the bacterial concentration both on the outer layer and middle layer of the mask increases with prolonged time, the survival bacteria concentration is far less than the counterpart of the commercial one. The much lower bacteria concentration on the g‐C_3_N_4_‐V_C_ Ns functionalized mask should be the result of the light‐triggered ROS generation on the g‐C_3_N_4_‐V_C_ Ns modified fabrics, as the ROS is highly toxic and biocidal to the pathogens. The unceasing generation of ROS on the outer layer of the mask can continue to inactivate the adhered bacteria, thus prolonging the protective time of the mask against bacteria aerosol. As a result, no surviving bacteria is tested after 5 h of bacteria protection in the inner layer of the mask under such a higher microorganism environment. It should be noted here that the pathogens aerosol concentration in the real environment is far less than the one used in this work,^[^ ^43^, ^44^ ^]^ which in turn means that the lifetime time of our constructed mask should be much longer than 5 h. Therefore, the prolonged protective lifetime can reduce the mask replacement frequency, leading to the largely medical waste decrement. Furthermore, the as‐prepared g‐C_3_N_4_‐V_C_ Ns is tested capable of excited by visible light (Figure S4, Supporting Information), suggesting that the as‐built photocatalytic respirator may not only work outdoors with sunlight but also works indoors where lamplight is accessible. The work demonstrated here opens new viewpoints in designing pathogens biocidal protective devices for light‐triggered long‐term health protection, which holds significant promise in specific environment self‐protection.

## 3 Conclusion

In summary, we have demonstrated the successful carbon vacancy‐mediated exciton dissociation over carbon nitride by using the combined methods of experimental and DFT calculations. Results show that carbon vacancy installation is capable of creating energy energy‐disordered landscape with buil‐in electric force higher than the exciton binding energy, leading to the high ROS generation as a result of the fast electron‐hole special separation. The carbon vacancy‐modified material is processed as a bio‐protective respirator for self‐protection with the functions of particle filtration and light‐triggered pathogens killing. The as‐prepared bio‐protective mask shows excellent long‐term light‐triggered self‐protection performance against pathogen aerosol and particles. The work demonstrated in this work provides a new viewpoint for extending the use of photocatalysts to air disinfection areas, which holds great potential in improving the self‐protection performance of traditional masks and reducing medical waste as a result of prolonged mask use time.

## 4 Experimental Section

### Materials Synthesis

Pristine carbon nitride (g‐C_3_N_4_ Ns) was fabricated via the direct polymerization of urea in a muffle furnace at 550 °C for 4 h. After natural cooling to room temperature, the resulting yellow powder was ground and collected. As for the synthesis of carbon vacancy‐modified carbon nitride (g‐C_3_N_4_‐V_C_ Ns), 0.5 g of the synthesized g‐C_3_N_4_ Ns was loaded in a porcelain boat without a cover. The container was put into a tube furnace and heated at 550 °C under water vapor conditions to facilitate the reaction between vapor and carbon atoms of carbon nitride, leaving carbon vacancies on the network. After natural cooling to room temperature, the brownish‐yellow powder was collected and donated as g‐C_3_N_4_‐V_C_ Ns.

### Carbon Nitride‐Based Respirator Fabrication

Using a spraying method, the carbon nitride functionalized respirators were fabricated by loading the materials onto the out‐layer nonwovens of the commercial respirator. Typically, 10 mg of catalyst was allowed to disperse in 10 mL of methanol, and the resulting dispersion was sprayed onto the non‐woven using a homemade sprayer. After drying at room temperature, the catalyst‐coated non‐woven was sewn onto the outside of the respirators. As a control, the commercial respirator was used as a reference without any modification.

### Materials Characterizations

Vapor at high temperatures would selectively etch the carbon atoms of the carbon nitride skeleton, which might lead to structure changes. In this regard, the morphology changes were first investigated by using S4800 scanning electron microscopy (SEM) and transmission electron microscopy (TEM, Tecnai G2F30S‐Twin). The chemical structures of as‐prepared materials were tested by using an X‐ray photo‐electron spectrometer (VGESCALAB250) equipped with a monochromater Al‐Kα source. In addition, the surface functional groups of the materials were uncovered by using a Bruker VERTEX 70 FTIR apparatus, during the tests, KBr was used as a reference. While for the crystal structure tests, a Samrtlab (9) Powder X‐ray diffraction (XRD) diffractometer equipped with Cu‐Kα radiation was employed. The introduction of vacancies may alert the optical and electronic properties of carbon nitride, here, the light adsorption properties were tested using a Shimadzu UV‐2450 spectrophotometer with bare BaSO_4_ as reference. The charge recombination behaviors were investigated using a Hitachi F‐7000 fluorescence spectrophotometer, and the excitation wavelength was set as 330 nm. Here, solid EPR was employed to verify the successful installation of carbon vacancies using a Bruker ERP machine (Bruker A300).

### Experimental Processes

As the materials were designed for health protection, here E‐coli and S‐aureus were selected as representative pathogens to evaluate the as‐prepared powder materials and functional respirators. First, E‐coli or S‐aureus was incubated in a liquid LB solution at 37 °C for 24 h to promote the bacteria reproduction. Afterward, the solution was centrifuged to separate the bacteria and redispersed into 0.9 wt.% saline to form a mixed bacteria solution, and the bacteria concentration of the solution was controlled to be ≈10^7^ or 10^6^ cfu mL^−1^. For the powder catalyst, photocatalytic activities were performed in a quartz container with flowing cooling water to maintain the reactor temperature ≈20 °C during the photocatalytic reactions. Furthermore, a 300 W Xenon lamp with an output light wavelength of λ > 420 nm served as the light source. Before the experiments, all containers and consumable materials were treated in an autoclave at 120 °C for 2 h to ensure sterility. In a typical procedure, 50 mg of catalyst was dispersed into the bacteria solution, after stirring in dark conditions for half an hour, the dispersion was subjected to light irradiation. At predetermined time intervals, 1 mL of solution was sampled using a syringe, diluted with saline, and sprayed onto the nutrient agar without removing the catalyst. The resultant agars were cultured at 37 °C for 24 h before counting the number of bacterial colonies.

As for the materials functionalized respirator, the catalytic activities were evaluated using a homemade reactor simulating the breathing process, during which the bacteria aerosol would go through the respirator (using an electric van). During the experiment, a Xenon lamp with output light of λ > 420 nm was used as simulated light to irradiate the respirator, triggering the bacteria inactivation processes. At the designed time, the respirator was washed with saline to transfer the bacteria on the respirator into the solution, and the resultant solution was sprayed on agar and cultured at 37 °C. After 24 h, the number of colonies was counted to get the bacteria concentration on the respirator.

### Analytic Methods

It was known that bacteria inactivation is highly correlated with the type and amount of generated reactive oxygen radicals (ROS). Therefore, it was necessary to clarify the type and quantify the amount of generated ROS. Since 3,30,5,50‐tetramethylbenzidine (TMB) was highly reductive and can be oxidized by all kinds of radicals including photo‐generated holes, to give a blue color, in this regard, TMB was then used as a probe to quantify the generated amount of ROS.^[^ ^15^, ^45^ ^]^ Typically, 10 mg of powder form catalyst was dispersed in 20 mL of ultrapure water and sonicated for 2 min to form a uniform dispersion. Then, 50 µL of the above suspension, 50 µL TMB (10 mm), and 2.5 mL of acetate buffer solution (NaAc/HAc with pH ≈four) were added into a quartz cell in turn. The resulting cell was subjected to light irradiation, and after being illuminated for a fixed time, the light absorbance at 651 nm of the solution in the cell was analyzed by using a UV–vis spectrophotometer (UV‐1800 PC) so as to uncover the TMB oxidation performance. Here, the higher the TMB oxidation performance, the more the generated ROS. The type of generated ROS was further clarified by using the radical capture experiments, where superoxide dismutase (SOD), L‐histidine, tertiary butyl alcohol (t‐BuOH), catalase, and EDTA‐2Na were respectively employed as probes to identify the generated reactive species of O_2_^·−^, ^1^O_2_, ~OH, H_2_O_2_, and photo‐induced holes. As a powerful tool to provide direct evidence of the generated radials in the catalytic system, electron paramagnetic resonance (EPR) was also employed to further confirm the generated type of ROS.

### Calculations

The electronic structure calculations for the g‐C_3_N_4_ surface were performed using the Vienna Ab initio Simulation Package (VASP). To accurately model the core electrons, projector augmented wave (PAW) potentials with the generalized gradient approximation (GGA) was employed using Perdew–Burke–Ernzerhof (PBE) exchange‐correlation functions. The calculation thresholds of the total energy convergence and the force convergence on each atom were 10^−6^ eV and 0.02 eV Å^−1^, respectively. The energy cutoffs for surface systems were set to 500 eV for the geometry optimization calculations. In addition, the most effective semi‐empirical DFT‐D3 method was introduced into the calculation system to correct the weak interaction.

## Conflict of Interest

The authors declare no conflict of interest.

## Supporting information

Supporting Information

## Data Availability

The data that support the findings of this study are available from the corresponding author upon reasonable request.
