# Supplementary material for: Bioprotective Respirator Assembled by Defective Carbon Nitride for Long‐Term Light Triggered Health Protection
Source: Adv Sci (Weinh). 2024 Jun 19;11(31):2403098. doi: 10.1002/advs.202403098 (PMC11336926; doi:10.1002/advs.202403098)
Supplement: Supplementary file 1 — Supporting Information [file ADVS-11-2403098-s001.docx]

**Bioprotective** **Respirator Assembled by Defective Carbon Nitride for Long-Term Light Triggered Health Protection**

***Zhenxing Zeng* ^a, b^*, Qi Zhang* ^a, b^*, Fei Ye* ^c^*, Xueming Dang* ^d^*, Xin Jiang* ^e^*, Guochun Lv* ^a,^ **, Xiaojing Wang* ^a, b^*, Hong Peng* ^a^*, Dexin Fang* ^a^*, Hong Xiao* ^a^*, Yanzong Zhang ^a^, Ganxue Wu* ^a^*, Jie Mao* ^f,^ **, Munir Ahmad ^g^, Shihuai Deng* ^a, b,^ *****

***^a^*** *College of Environmental Sciences, Sichuan Agricultural University, Chengdu 611130, People’s Republic of China.*

***^b^*** *Sichuan Provincial Engineering Center of Agricultural Environmental Pollution Control, Chengdu 611130, China.*

***^c^*** *Hebei Key Laboratory of Applied Chemistry, School of Environmental and Chemical Engineering, Yanshan University, Qinhuangdao 066004, China.*

***^d^*** *Key Laboratory of Industrial Ecology and Environmental Engineering (Ministry of Education), School of Environmental Science and Technology, Dalian University of Technology, Dalian, 116024, China.*

***^e^*** *School of Environment and Energy, Jiangxi Modern Polytechnic College, Nanchang, 330095, China.*

***^f^*** *Research Center for Eco-Environmental Sciences, Chinese Academy of Sciences, Beijing 100085, China.*

***^g^*** *Institute of Carbon Neutrality, Zhejiang Wanli University, Ningbo 315100, China.*

* To whom correspondence should be addressed:

**E-mail:** [lgcttxs@foxmail.com](mailto:lgcttxs@foxmail.com)

**E-mail:** [jiemao@rcees.ac.cn](mailto:jiemao@rcees.ac.cn)

**E-mail:** [shdeng8888@163.com](mailto:hdeng8888@163.com)

**Table S1**. Elemental contents of C, N for g-C_3_N_4_ Ns and g-C_3_N_4_-V_C_ Ns.


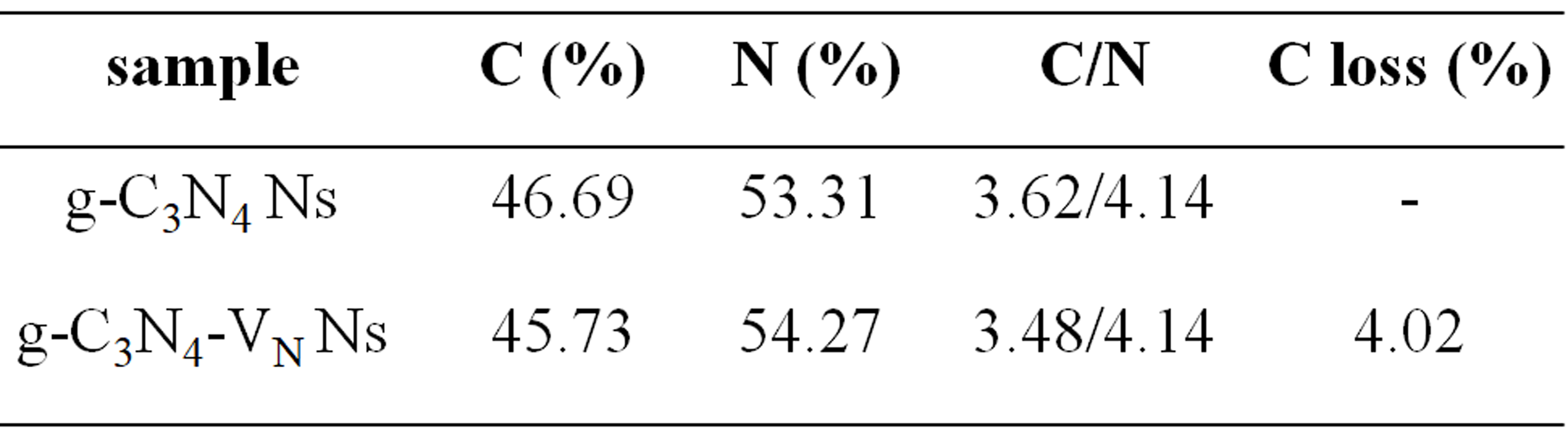


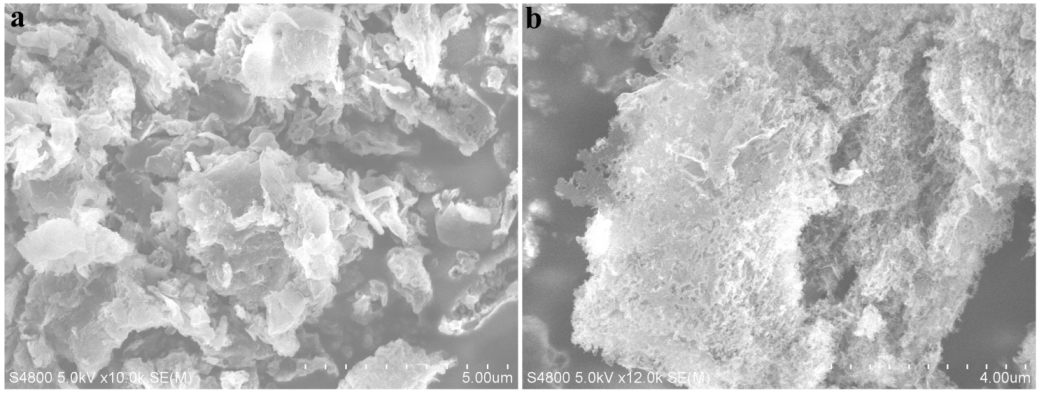


**Figure S1.** SEM images of (a) g-C_3_N_4_ Ns and (b) g-C_3_N_4_-V_C_ Ns.


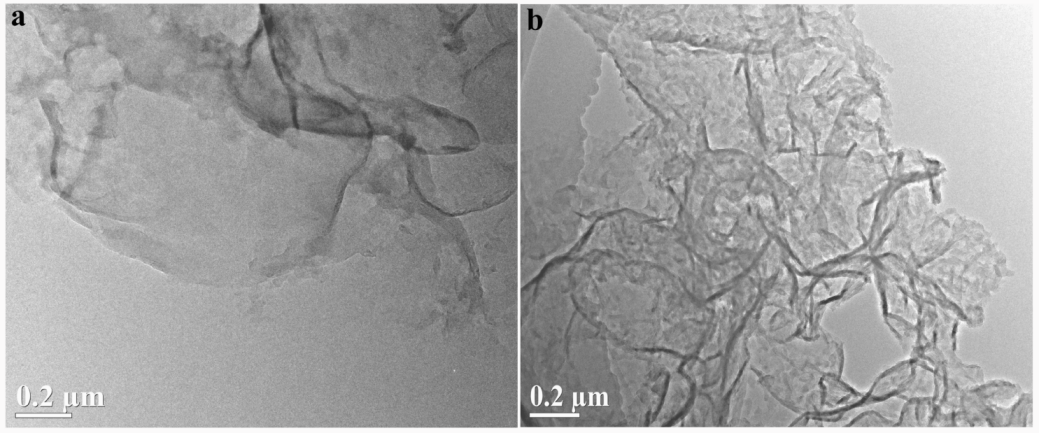


**Figure S2.** TEM images of (a) g-C_3_N_4_ Ns and (b) g-C_3_N_4_-V_C_ Ns.


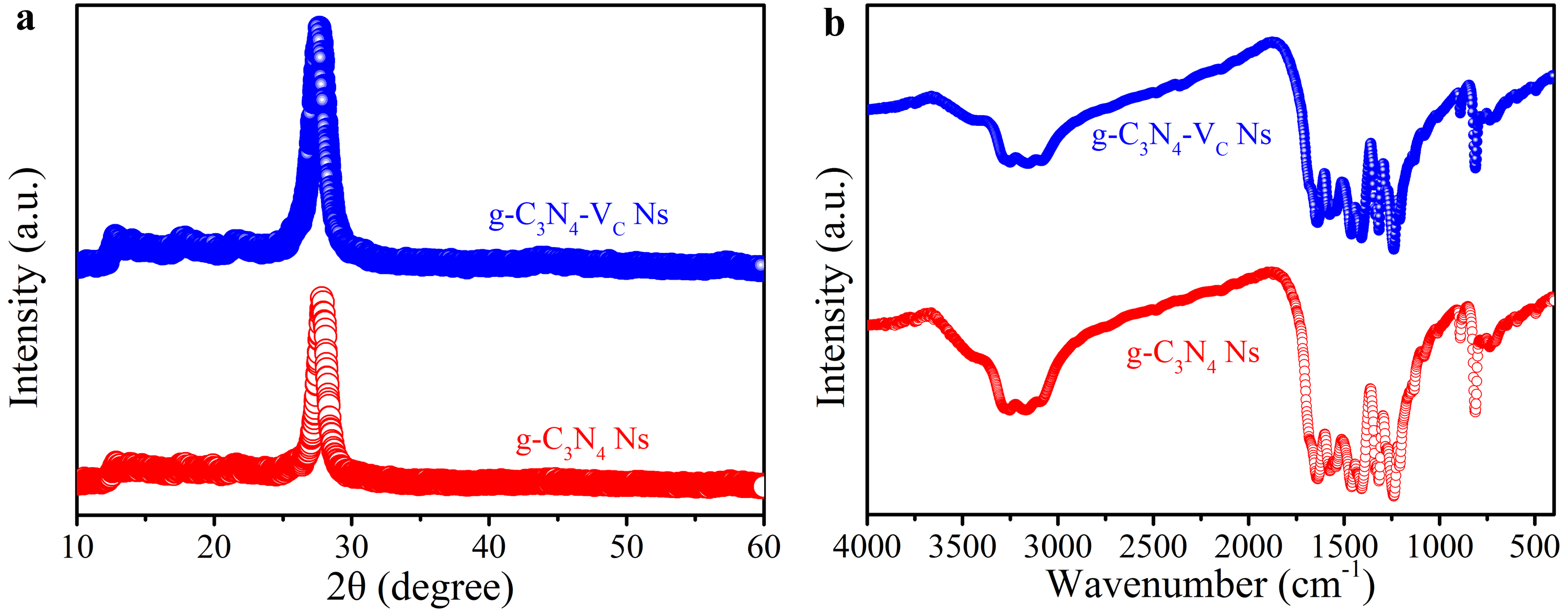


**Figure S3.** (a) XRD patterns and (b) FT-IR spectra of g-C_3_N_4_ Ns and (b) g-C_3_N_4_-V_C_ Ns.


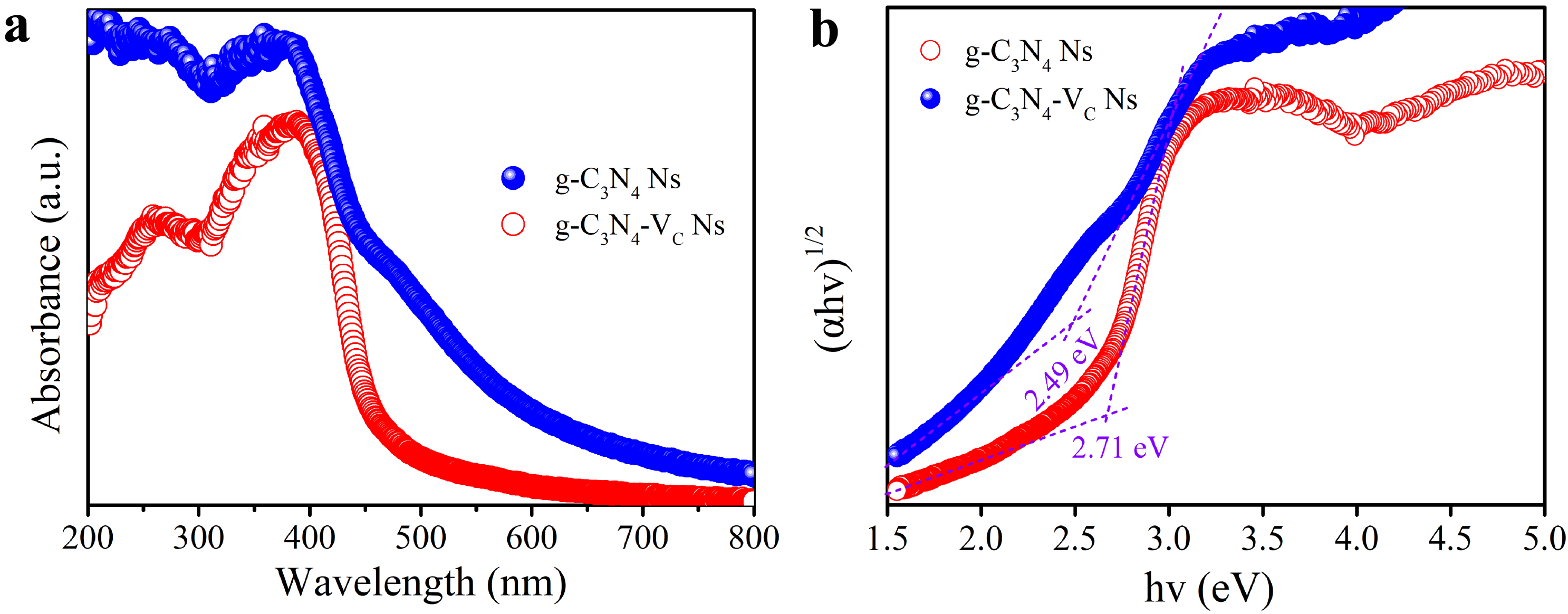


**Figure S4.** (a) UV-Vis spectra and (b) the corresponding tauc plots of g-C_3_N_4_ Ns and g-C_3_N_4_-V_C_ Ns.


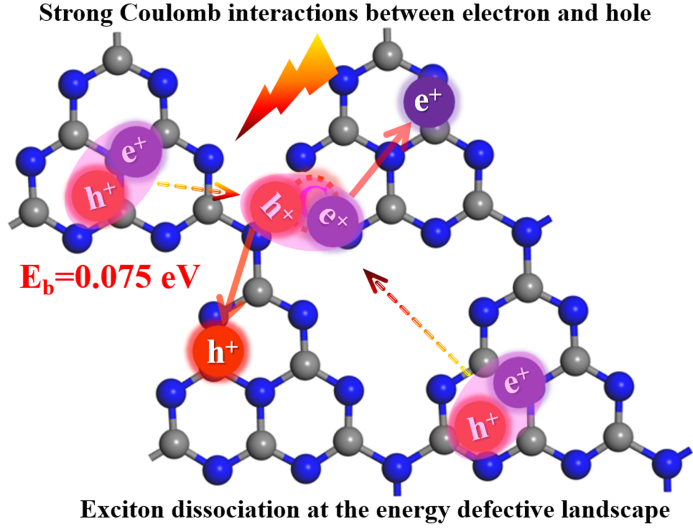


**Figure S5.** Illustration of carbon vacancy mediated exciton dissociation.

**
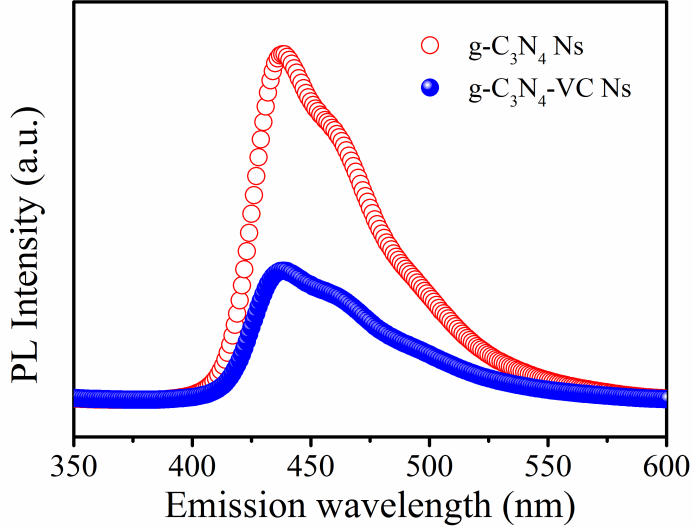
**

**Figure S6.** PL spectra of g-C_3_N_4_ Ns and (b) g-C_3_N_4_-V_C_ Ns.


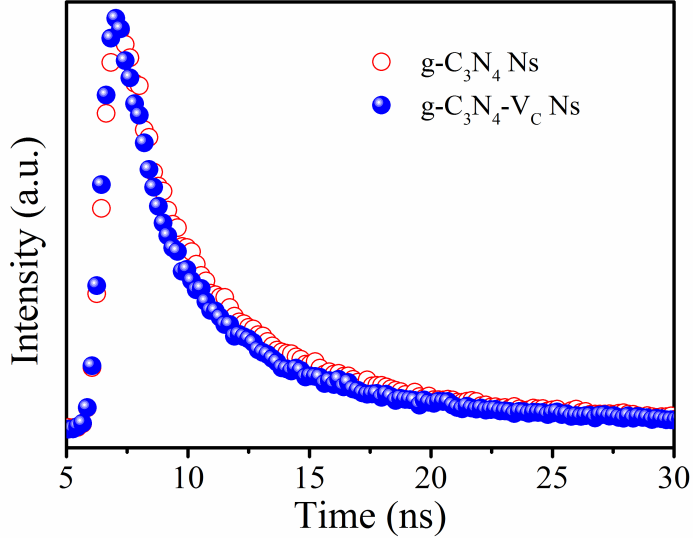


**Figure S7.** Time resolved PL spectra of g-C_3_N_4_ Ns and (b) g-C_3_N_4_-V_C_ Ns.

**Table S2**. Detailed TR-PL interpretation parameters.

| **Sample τ_1_/ns τ_2_/ns τ_3_/ns Ave. τ_1_/ns** | | |
| --- | --- | --- |
| **g-C_3_N_4_ Ns** | 1.000 4.532 18.090 | 7.57 |
|  | 16.74% 55.19% 27.08% |  |
| **g-C_3_N_4_-V_C_ Ns** | 0.5543 3.5016 15.3558 | 6.80 |
|  | 14.14% 54.51% 31.35% |  |


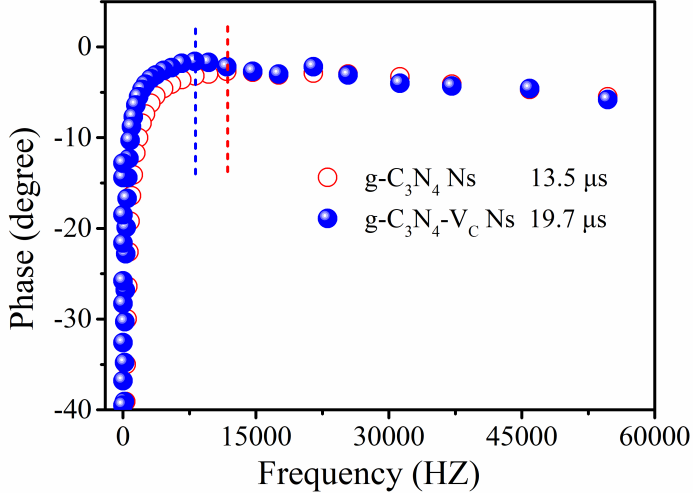


**Figure S8.** Bode spectra of g-C_3_N_4_ Ns and (b) g-C_3_N_4_-V_C_ Ns.

**
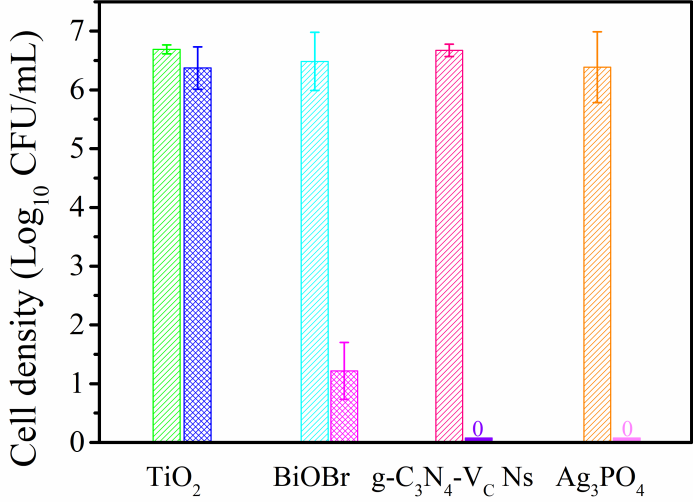
**

**Figure S9.** Survival E-coli cells on the out layer of TiO_2_, BiOBr, g-C_3_N_4_-V_C_ Ns, and Ag_3_PO_4_ functionalized respirators after 45 min visible light irradiation using.


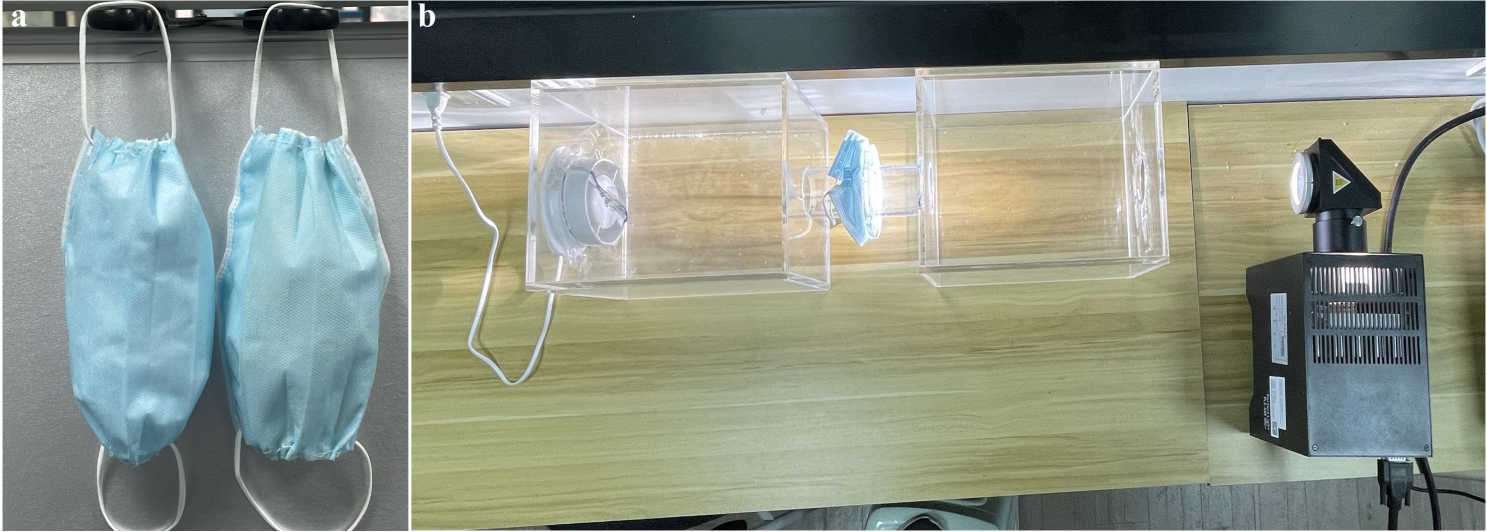


**Figure S10**. Digital pictures of (a) functionalized mask (left for the commercial mask, and the right for the g-C_3_N_4_-V_C_ Ns loaded mask), (b) homemade reactor simulating the breathing process.

**
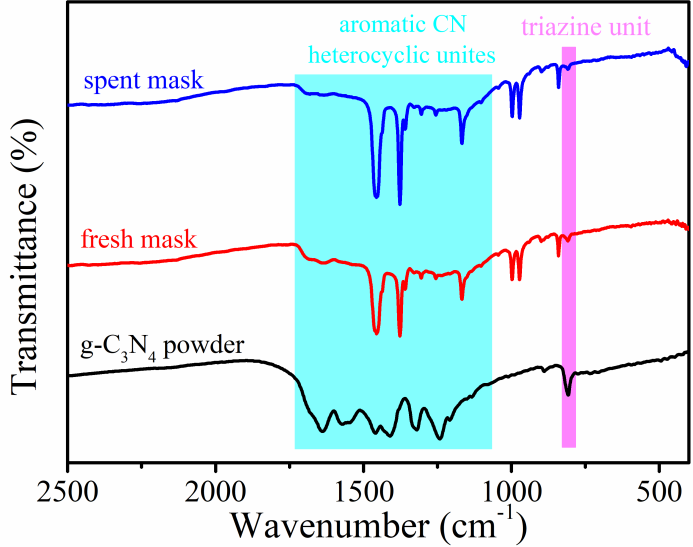
**

**Figure S11.** FT-IR spectra of the modified non-woven before and after bacteria inactivation tests.
